# Mechanics of biofilms formed of bacteria with fimbriae appendages

**DOI:** 10.1371/journal.pone.0243280

**Published:** 2020-12-08

**Authors:** Xing Jin, Jeffrey S. Marshall

**Affiliations:** Department of Mechanical Engineering, University of Vermont, Burlington, VT, United States of America; University of Notre Dame, UNITED STATES

## Abstract

Gram-negative bacteria, as well as some Gram-positive bacteria, possess hair-like appendages known as fimbriae, which play an important role in adhesion of the bacteria to surfaces or to other bacteria. Unlike the sex pili or flagellum, the fimbriae are quite numerous, with of order 1000 fimbriae appendages per bacterial cell. In this paper, a recently developed hybrid model for bacterial biofilms is used to examine the role of fimbriae tension force on the mechanics of bacterial biofilms. Each bacterial cell is represented in this model by a spherocylindrical particle, which interact with each other through collision, adhesion, lubrication force, and fimbrial force. The bacterial cells absorb water and nutrients and produce extracellular polymeric substance (EPS). The flow of water and EPS, and nutrient diffusion within these substances, is computed using a continuum model that accounts for important effects such as osmotic pressure gradient, drag force on the bacterial cells, and viscous shear. The fimbrial force is modeled using an outer spherocylinder capsule around each cell, which can transmit tensile forces to neighboring cells with which the fimbriae capsule collides. We find that the biofilm structure during the growth process is dominated by a balance between outward drag force on the cells due to the EPS flow away from the bacterial colony and the inward tensile fimbrial force acting on chains of cells connected by adhesive fimbriae appendages. The fimbrial force also introduces a large rotational motion of the cells and disrupts cell alignment caused by viscous torque imposed by the EPS flow. The current paper characterizes the competing effects of EPS drag and fimbrial force using a series of computations with different values of the ratio of EPS to bacterial cell production rate and different numbers of fimbriae per cell.

## Introduction

In bacterial biofilms, bacteria are enmeshed in a self-secreted extracellular polymeric substance (EPS), which is permeated by an aqueous solvent that transports nutrients, minerals and other chemicals through the EPS [1]. In general, bacteria absorb nutrients and water, using them to grow and to produce EPS. [The water within the biofilm exists in a bound state (i.e., water of hydration associated with the EPS) or in a free state that can flow through the biofilm. For modeling purposes, we regard the former as part of the EPS, and use the term 'water' to refer to water in the latter (free) state.] Bacterial biofilms are important in water treatment processes [2], in environmental processes such as production of greenhouse gases from the soil [3], in biofouling of ships and marine structures [4], and in food processing [5, 6]. Biofilms are responsible for the majority of human infectious diseases [7, 8], particularly in post-surgical infections or chronic infections.

A key feature that enables adhesion of bacterial cells both to each other and to other surfaces is the short hair-like appendages called fimbriae (singular fimbria), which are found on most Gram-negative bacteria and on some Gram-positive bacteria [9–11]. (These appendages are also referred to in some literature as pili or attachment pili). There are on order of 1000 fimbriae on a single cell, each 3–10 nm thick and 1–5 μm long. At the microstructural level, a single fimbria appendage has the form of a coiled helix-shaped protein (pilin), with sticky proteins (adhesins) located on the fimbria tip. The adhesin proteins bind to receptors on other bacteria or on host cells using a 'catch-bond' mechanism, in which the adhesive force becomes stronger (up to a limit) as the tension force acting on a fimbria is increased [12, 13]. Once attached, a fimbria can stretch to several times its original length. Experiments characterizing the stress-strain behavior of individual fimbria were reported by Chen et al. [14] and Forero et al. [15].

Numerous experimental studies have demonstrated that different types of fimbriae play a critical role in enabling certain bacteria to form biofilms, although the enhancement of bacterial attachment ability and biofilm growth is dependent on both the type of bacteria and the type of fimbriae [16]. For instance, type 3 fimbriae were found to strongly promote biofilm formation for *Klebsiella pneumoniae* [17–20]. Bak et al. [21], Zuberi et al. [22], and Lasaro et al. [23] showed that biofilm formation in *Escherichia coli* is inhibited when type 1 fimbriae are suppressed. Rodrigues and Elimelech [24] and Wang et al. [25] examined role of type 1 fimbriae for biofilm formation of *E*. *coli*, with fimbriaed, non-fimbriaed and wild bacteria. They found that type 1 fimbriae are not necessary for initial reversible cell attachment, but that they are necessary for irreversible cell attachment and subsequent biofilm development. Cohen et al. [26] showed that presence of fimbriae enhances aggregation of *E*. *coli* with small clay particles. McLay et al. [27] gradually varied the degree of fimbriation (by varying the number of fimbriae attached to the cells), and showed that the ability of cells to adhere gradually decreases as the degree of fimbriation is decreased.

Understanding the dynamics of biofilm systems is challenging because of the large number of parameters involved and the highly nonlinear, complex dynamics exhibited by biofilm systems. Mathematical modeling allows investigators to easily activate and deactivate different biofilm features to gain insight into their impact on the system [28, 29]. Both discrete and continuum models have been developed and applied to biofilm systems, both with different advantages and disadvantages [30–38]. Continuum models treat bacteria, EPS and water as interacting continua, for each of which there is an associated continuous concentration and velocity field and related mass and momentum conservation equations [35–37, 39–41]. Discrete models treat biofilms as a collection of individual ‘agents’ (or particles) that interact with each other, with the surface to which the biofilm is attached, and with other surrounding biofilm components (such as EPS and water). With discrete models, it is a simple matter to assign properties, shapes, and behaviors to individual bacteria and then allow the model to determine how these characteristics lead to different collective (emergent) behavior of the biofilm system [31–34, 38, 42, 43]. However, most continuum models do not account for the numerous forces acting between individual bacterial cells, whereas most discrete models (also known as *individual based models*) do not account for the separate flow fields of water and EPS past the cells. Both of these types of models tend to over-simplify the cell interaction forces, often omitting important forces for the biofilm dynamics. A new type of hybrid model was recently developed by the current investigators [44] which surmounts many of these objections. The model uses a discrete approach to follow motion and interaction of individual bacterial cells while using a continuum approach to model EPS, water and nutrient transport around and within the biofilm, including absorption of nutrients and water and EPS production by the bacteria. The continuum model is based on an extension of that of Cogan and Keener [39], with an improved model for the water-EPS interfacial force. The discrete model is based on an extension of an accurate discrete element method (DEM) for adhesive particle flows [45–47], and includes a wide range of cell-EPS and cell-cell forces and torques for both spherical [31] and spherocylindrical cell shapes [34, 42, 43, 48, 49].

The current paper extends the hybrid model of Jin et al. [44] to include fimbrial force and non-spherical bacterial cells, and then uses this extended model to examine the influence of fimbrial force and EPS flow on biofilm growth processes. We argue that of the many different forces present, the fimbriae tension and the EPS drag force dominate in determining the structure of the bacterial colony as it develops within the biofilm. The method section gives an overview of the biofilm growth model used in the study, including the continuum models for EPS and water transport and the discrete model for the bacterial cells. The results of the paper include an examination of the effects of varying the ratio of EPS to cell production rates and the number of fimbriae attached to each cell. Conclusions are given in the last section.

## Computational method

### Discrete model

The biofilm mechanics were simulated using a hybrid computational model in which bacterial cells are represented by spherocylindrical particles and the flow of water, EPS and nutrients are computed as continua on a grid that spans the flow field [44]. Spherocylinders are formed of cylindrical bodies with hemispherical end caps. The cell minor semi-axis *b* is set equal to the cylinder radius, and the major semi-axis *a* is equal to half the cylinder length plus the radius of the hemispherical end-cap. The bacterial cell motion was computed by solving the individual cell momentum and angular momentum equations at equilibrium or

$$F_{BE}+F_{BB}=0,\;M_{BE}+M_{BB}=0,$$ (1)

where **F**_*BE*_ and **M**_*BE*_ denote forces and torques between the bacterial cells and the surrounding EPS and **F**_*BB*_ and **M**_*BB*_ denote forces and toques between two or more bacterial cells, or between a bacterial cell and a wall. The cell inertia is neglected since the Stokes number is several orders of magnitude less than unity for this problem. The two most important EPS-bacteria interaction forces contained in **F**_*BE*_ are the drag force **F**_*d*_ and the lubrication force $F_{l}$. The Weissenberg number, which is given by We $=\dot{\gamma }\lambda$ where $\dot{\gamma }$ is the shear rate and *λ* is the material relaxation time, is We ≅0.1 for this problem. This estimate is based on a growth time scale of *T*≅3 hrs [28, 50] with $\dot{\gamma }≅1/T$, and a relaxation time of *λ* = 18 min, which is nearly constant for different types of biofilms [51]. A study of drag forces on spherical particles in a low Reynolds number, viscoelastic fluid [52] concludes that the drag on the particle can be well approximated by the Stokes law for We ≤0.1. Therefore, the drag force **F**_*d*_, and the associated viscous torque **M**_*d*_, can be approximated using the well-established theory for low Reynolds number flow past ellipsoidal particles [53–55]. Details of the force and torque expressions can be found in Chesnutt and Marshall [56].

The lubrication force $F_{l}=F_{l}n$ is caused not only by relative motion between the particle centers, but also by cell growth and EPS production. An expression for lubrication force magnitude that accounts for these different effects is obtained as

$$F_{l}=-6\pi \mu_{E}R^{2}\left(\frac{\dot{h}+U_{LE}}{h+\delta }\right),$$ (2)

where *μ*_*E*_ is the EPS viscosity, *h* is the closest separation distance between the cell surfaces, *δ* is a constant gap width between cell surfaces at collision, *U*_*LE*_ is the sum of the normal EPS velocity at the contact point relative to the surface velocity for each particle of a colliding pair, and *R* is the effective radius of curvature at the collision point.

The most important cell-cell interaction forces contained in **F**_*BB*_ are the elastic rebound force, the cell surface adhesion force, and the fimbrial force **F**_*f*_ = *F*_*f*_**n**. The first two of these forces are nonlinearly coupled to form a single surface collision/adhesion force **F**_*sc*_, an expression for which is given by the classical Johnson-Kendell-Roberts (JKR) theory [57]. These various effects can be combined to write the EPS-bacteria and bacteria-bacteria interaction forces and torques as

$$F_{BE}=F_{d}+F_{l}n,\;M_{BE}=F_{l}r_{i}\times n+M_{d}.$$ (3)

$$F_{BB}=F_{sc}n+F_{f}n,\;M_{BB}=(F_{sc}+F_{f})r_{i}\times n.$$ (4)

Here, **n** is the unit normal vector of the particle *i* at the contact point C, and **r**_*i*_ is the vector that extends from the center of particle *i* to the contact point.

The fimbrial force exerts a tension between cells when the fimbriae from one cell attach to the surface of another cell, and the two cell surfaces are pulled apart by an external force. Since tracking the attachment and stretch of individual fimbriae for a large number of cells would necessitate a very large computational expense, we instead adopt an approximate model in which it is assumed that the fimbriae of each cell have a uniform unstretched length *h*_*f*0_ and a uniform fimbriae number density *n*_*f*_ (defined as number of fimbriae per unit area). A spherocylindrical fimbriae capsule is assumed to surround each cell with semi-major axis *a*+*h*_*f*0_ and semi-minor axis *b*+*h*_*f*0_. The number *N*_*f*_ of attached fimbriae between two nearby cells is therefore equal to the fimbriae number density *n*_*f*_ times the attachment area *A*_*a*_ on the fimbriae capsule, or

$$N_{f}=A_{a}n_{f}.$$ (5)

The attachment area *A*_*a*_ is defined as the area on the fimbriae capsule of one cell that intersects the surface of another cell. The algorithm for determination of fimbriae connections to cell surfaces and calculation of the attachment area is similar to that in Ref. [58, 59]. The magnitude of the fimbrial force *F*_*f*_ is related to the number of connected fimbriae by

$$F_{f}=N_{f}T_{f},$$ (6)

where *T*_*f*_ is the average tension of a single fimbria appendage attached between the two cells. It is assumed that the fimbrial force acts along a line that is orthogonal to, and passes through the center of, the attachment area *A*_*a*_.

**Fig 1:**
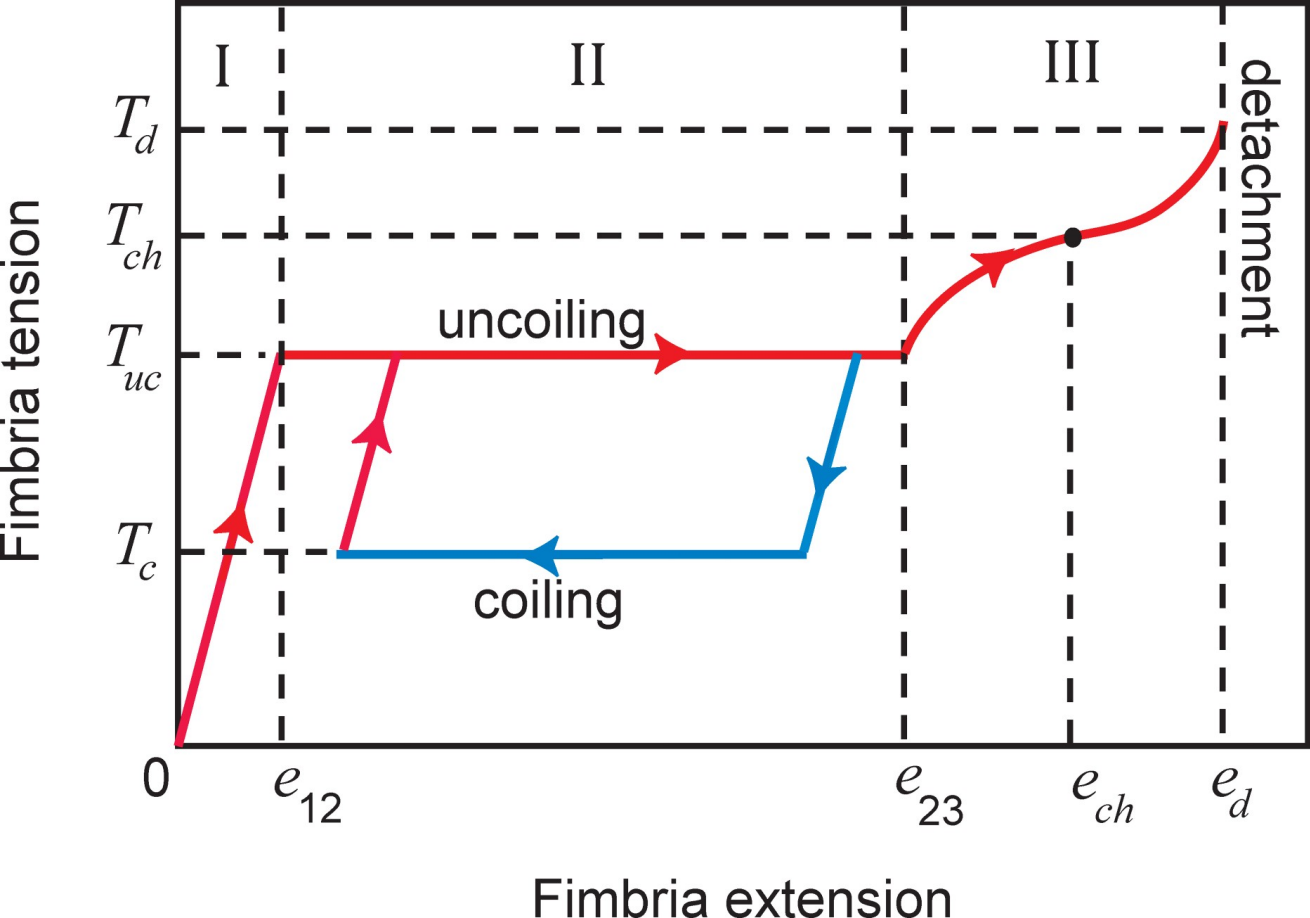
Typical force-extension plot for a single fimbria appendage. The regions of fimbria extension are identified by Roman numerals at the top of the plot. The force-extension curve of a single fimbria in tension between two surfaces that are being pulled apart is identified by a red curve. The blue curve is the force-extension curve for a case where the direction of relative motion between the surfaces is reversed at a specified time and the surfaces are pushed back toward each other.

Experiments using an atomic force microscope [14, 15] have shown that fimbria tension *T*_*f*_ depends on both the direction of relative motion of the two attached surfaces and on the fimbria extension from its equilibrium length. An idealized force-extension curve for a single fimbria that is characteristic of the experimental data is plotted in Fig 1. The fimbria tension is characterized by three different regimes, labeled regions I, II and III in the figure. In region I, the fimbria stretches while retaining its helical form, resulting in a force-extension response similar to Hooke's law for a linear elastic medium with a Young's modulus *E*_*f*_. In region II, the helical fimbria begins to uncoil as a result of stretching, which results in a constant tension force *T*_*uc*_ that is independent of fimbria extension. This region of the force-extension curve can continue for extensions out to several micrometers, or several times the fimbria length. Region III occurs once the fimbria is fully uncoiled to form a thin filament. In region III, the fimbria tension increases rapidly up to a point of maximum extension *e*_*d*_, at which the tension has the value *T*_*d*_. The fimbria detaches from the cell surface when stretched at extensions beyond *e*_*d*_, and *T*_*d*_ and *e*_*d*_ are correspondingly called the detachment tension and extension. The fimbria tension and extension at the inflection point of the force-extension curve in region III are called the characteristic tension *T*_*ch*_ and characteristic extension *e*_*ch*_, respectively.

The blue line in Fig 1 shows the force-extension curve in the event that the relative motion between the two cells were reversed at a time while the fimbria was in the uncoiling region (region II). In this case, the force-extension curve traces a different path, as the fimbria begins to recoil itself to reform its helical structure. The fimbria tension consequently drops down (following a line parallel to the elastic tension line in region I) to a second constant level, called the coiling tension *T*_*c*_. If at some point the direction of relative motion of the two cells is again reversed such that they again move apart from each other, the tension would increase back up to the uncoiling tension *T*_*uc*_ following a line that is parallel to the elastic tension line in region I (shown by a red line in Fig 1).

Approximate mathematical expressions for the fimbria tension when the surfaces are moving apart from each other (i.e., when $\dot{h}>0$) are given by

$$T_{f}=\{\begin{array}{c} E_{f}(h-h_{f0})\\ T_{uc}\\ T_{ch}+C\sinh[D(h-e_{ch})] \end{array}\begin{array}{l} \;\mathrm{in}\;\mathrm{region}\;I\;(0\leq h\leq e_{12})\\ \;\mathrm{in}\;\mathrm{region}\;\mathrm{II}\;(e_{12}\leq h\leq e_{23})\\ \;\mathrm{in}\;\mathrm{region}\;\mathrm{III}\;(e_{23}\leq h\leq e_{d}) \end{array}$$ (7)

where the coefficients *C* and *D* are determined by solving the set of nonlinear equations

$$T_{ch}+C\sinh[D(e_{23}-e_{ch})]=T_{uc},$$ (8A)

$$T_{ch}+C\sinh[D(e_{d}-e_{ch})]=T_{d}.$$ (8B)

If the direction of motion of the attached surfaces is reversed at a separation distance *h* = *h*_*rev*_ in region II (such that $\dot{h}<0$), the fimbriae tension is alternatively given by

$$T_{f}=\{\begin{array}{c} T_{uc}-E_{f}(h_{rev}-h)\\ T_{c} \end{array}\begin{array}{l} \;\mathrm{for}\;h_{rev}-h<(T_{uc}-T_{c})e_{12}/T_{uc}\\ \;\mathrm{for}\;h_{rev}-h\geq (T_{uc}-T_{c})e_{12}/T_{uc} \end{array},$$ (9)

which corresponds to the blue line in Fig 1. Typical values of these critical extensions and tensions are listed in Table 1.

**Table 1:** Typical values of fimbria extensions and tensions.

Fimbria extension	Length (μm)	Fimbria tension	Force (pN)
*e*_12_	1.5	*T*_*c*_	2.5
*e*_23_	2.5	*T*_*uc*_	6
*e*_*ch*_	3	*T*_*ch*_	9.5
*e*_*d*_	3.5	*T*_*d*_	13

Bacterial cells absorb water and nutrients and use these materials to grow and to produce EPS. For the current paper, we assume that all cells are of the same species and share the same material properties and follow the same size-dependent rule to grow and divide. Each bacterial cell produces new cell mass at a rate ${\dot{M}}_{B}$ and produces EPS at a rate ${\dot{M}}_{E}$. If *V*_*B*_ denotes the cell volume as a function of time, then

$$\frac{d}{dt}V_{B}(t)=\frac{{\dot{M}}_{B}}{\rho },$$ (10)

where *ρ* is the fluid density. A Monod model [60, 61] was used to specify cell growth rate as a function of the nutrient concentration *c*_*S*_. Since both water and nutrients are necessary for cells to grow and to produce EPS, we employed an extended form of the Monod model of the form

$${\dot{M}}_{B}={\dot{M}}_{B0}\left(\frac{c_{S}}{K_{S}+c_{S}}\right)\left(\frac{\alpha_{W}}{K_{W}+\alpha_{W}}\right),\;{\dot{M}}_{E}={\dot{M}}_{E0}\left(\frac{c_{S}}{K_{S}+c_{S}}\right)\left(\frac{\alpha_{W}}{K_{W}+\alpha_{W}}\right),$$ (11)

where *K*_*S*_ and *K*_*W*_ are half-saturation constants and ${\dot{M}}_{B0}$ and ${\dot{M}}_{E0}$ are the maximum bacteria and EPS growth rates. The last term in these equations typically has small effect, except in regions where water becomes scarce due to rapid EPS production and cell growth. Related extensions of the Monod model are discussed in more detail by Gonzo et al. [62] and Legner et al. [63]. Typical ranges of values for these coefficients were recorded by Picioreanu et al. [64] and Melaugh et al. [65] for different bacterial species.

When the cell volume *V*_*B*_(*t*) exceeds a critical value *V*_*B*,*crit*_, the cell divides to create two offspring cells with volumes *V*_1_ and *V*_2_, given by

$$V_{1}=V_{par}\left(\frac{1+\zeta }{2}\right),\;V_{2}=V_{par}\left(\frac{1-\zeta }{2}\right),$$ (12)

where *ζ* is a small random number with uniform distribution over the range (0,*ζ*_max_) and *V*_*par*_ is the volume of the parent cell prior to division. Cell division was performed using an algorithm (similar to [66]) that gradually moved the particles apart over a series of time steps until they were separated, and then released them to move according to their individual dynamics.

### Continuum model

The transport of water, EPS and nutrients in the biofilm are all computed using continuum equations on a grid spanning the biofilm computational domain. Communication between the discrete particles representing the cells and the continuum grid is an important part of the hybrid computational method. For instance, in solving the particle momentum equation, the EPS velocity at the cell centroid location is obtained by linear interpolation from the grid cell values in the continuum model. Similarly, it is necessary to homogenize the discrete data from the bacterial cell simulation in order to obtain values of corresponding averaged variables on the grid nodes of the continuum model. This homogenization procedure was used to obtain the particle concentration field *α*_*B*_, mass source fields per unit volume ${\dot{m}}_{B}$, ${\dot{m}}_{E}$ and ${\dot{m}}_{S}$ for the bacterial, EPS and nutrients, and interfacial body force per unit mass **f**_*BE*_. In the current paper, homogenization was performed using the conservative blob homogenization procedure described in Marshall and Sala [67], which is both discretely conservative and produces smooth fields with minimal noise. Considering the bacteria concentration field *α*_*B*_ as an example, in this homogenization scheme we write

$$\alpha_{B}(x,t)=\sum_{n=1}^{N}A_{n}(t)f(x‐x_{n},R_{n}),$$ (13)

where **x**_*n*_(*t*) is the position of the *n*th bacterial cell and *R*_*n*_ is the 'blob radius' used for smoothing the homogenization scheme. The function *f*(**x**-**x**_*n*_,*R*_*n*_) is selected as a smooth function, such as a Gaussian, whose integral over all space is unity. The coefficient *A*_*n*_ is related to the volume *V*_*n*_(*t*) of particle *n* and the grid cell volume *V*_*cell*_ by

$$A_{n}(t)=\frac{V_{n}(t)}{V_{cell}\sum_{i=1}^{N_{grid}}f(x_{i}‐x_{n},R_{n})},$$ (14)

which ensures that the homogenization scheme is discretely conservative.

Mass conservation of the EPS, water and nutrients is controlled by the following equations [39, 40]:

$$\frac{\partial \alpha_{E}}{\partial t}+\nabla \cdot (\alpha_{E}u_{E})={\dot{m}}_{E}/\rho ,$$ (15)

$$\frac{\partial \alpha_{W}}{\partial t}+\nabla \cdot (\alpha_{W}u_{W})={\dot{m}}_{W}/\rho ,$$ (16)

$$-\nabla \cdot \left[(\alpha_{W}+\alpha_{E})D_{S}\nabla c_{S}\right]={\dot{m}}_{S}.$$ (17)

where *α*_*W*_ and *α*_*E*_ are the volume concentrations of water and EPS, ${\dot{m}}_{W}$ is the mass source per unit volume of water, and **u**_*W*_ and **u**_*E*_ are the water and EPS velocity vectors. The nutrient mass concentration *c*_*S*_ are solved by the equilibrium diffusion Eq (17) subject to a nutrient mass source per unit mass ${\dot{m}}_{S}$ and diffusion coefficient *D*_*S*_. This equation neglects the time derivative and convection terms since the nutrient diffusion time scale (~1–2 min) is small compared to the bacterial division time scale *T* (~1 hr) [39, 68]. The volume fraction and the mass source terms are subject to the constraints

$$\alpha_{W}+\alpha_{E}+\alpha_{B}=1,$$ (18)

$${\dot{m}}_{E}+{\dot{m}}_{W}+{\dot{m}}_{B}+{\dot{m}}_{S}=0.$$ (19)

The momentum transport equations for water and EPS, respectively, are given by

$$-\alpha_{W}\nabla p+f_{WE}=0,$$ (20)

$$-\alpha_{E}\nabla p+\alpha_{E}\nabla \cdot \tau_{E}+f_{BE}-f_{WE}-\nabla \psi (\alpha_{E})=0,$$ (21)

where *p* is the pressure. Eq (20) balances the pressure gradient acting on the water with the water-EPS interfacial force per unit volume **f**_*WE*_. Inertia and friction terms within the water phase are neglected. In (21), *ψ*(*α*_*E*_) is the osmotic pressure (sometimes called ‘swelling pressure’)[69, 70] and **f**_*BE*_, which contains drag and lubrication forces, is the homogenized body force per unit volume between bacteria and EPS. The viscous term was retained in (21) since the EPS has much larger viscosity than water [51]. The bacterial division time scale is much longer than the biofilm elastic relaxation time (~18 min) [51], so the viscoelastic effects of the biofilm were neglected, and the EPS shear stress was given by the Newtonian expression

$$\tau_{E}=\mu_{E}[\nabla u_{E}+{\left(\nabla u_{E}\right)}^{T}]-\frac{2}{3}\mu_{E}(\nabla \cdot u_{E})I.$$ (22)

We note that the EPS velocity is not divergence-free, and so the ∇⋅**u**_*E*_ term must be retained in the shear stress expression (22). An expression for the water-EPS interaction force **f**_*WE*_ was proposed by Jin et al. [44] as

$$f_{WE}=A\alpha_{W}^{2}\alpha_{E}^{3/2}(u_{E}-u_{W}),$$ (23)

based on experimental results for permeability of water in hydrogels [71–73]. The interaction coefficient *A* is proportional to the ratio *μ*_*W*_/*ξ*^2^, where *μ*_*W*_ is the water viscosity and *ξ* denotes the pore size of the EPS hydrogel [41].

Eq (15) was solved over the entire computational domain (including within and outside the biofilm) by addition of a small diffusion term [39] as

$$\frac{\partial \alpha_{E}}{\partial t}+\nabla \cdot (\alpha_{E}u_{E})={\dot{m}}_{E}+D_{E}\nabla^{2}\alpha_{E},$$ (24)

where *D*_*E*_ is the EPS diffusion coefficient. This equation was solved using the Crank-Nicholson method for the diffusive term and second-order upwind differencing for the convective term. Eqs (20), (21) and (23) can be rearranged to obtain elliptic partial differential equations for **u**_*E*_ and *p*, which were solved using the Full Multigrid (FMG) algorithm [74–77], using the boundary conditions listed in Table 2. Once **u**_*E*_ and *p* are known, the water velocity field **u**_*W*_ was obtained from (20) and (23) as

$$u_{W}=u_{E}-\frac{1}{A\alpha_{W}\alpha_{E}^{3/2}}\nabla p.$$ (25)

**Table 2:** Boundary conditions in continuum variables.

Parameter	*x*	*y*	*z*
*α*_*E*_	periodic	zero gradient at bottom	periodic
		zero gradient at top	
**u**_*E*_	periodic	no slip at bottom (**u**_*E*_ = 0)	periodic
		zero gradient at top	
*p*	periodic	zero gradient at bottom	periodic
		constant at top (*p* = 0)	
*c*_*S*_	periodic	zero gradient at bottom	periodic
		constant at top (*c*_*S*_ = *c*_0_)	

## Results and discussion

The computations were performed in a cubic domain with 128^3^ grid points and side length *L* = 100 μm. The computational domain extends in the horizontal directions from (−0.5,0.5) in *x*/*L* and *z*/*L* and in the vertical direction from (0,1) in *y*/*L*. The continuum equations were solved using a 'fluid' time step Δ*t*_*f*_ = 100 *s*, and a multiple time-step procedure [45] was used for solution of the discrete equations with particle time step size Δ*t*_*p*_ = Δ*t*_*f*_/50 and collision time step size Δ*t*_*c*_ = Δ*t*_*p*_/50. A set of typical ranges and nominal values of a wide variety of parameters for bacterial biofilms is summarized in S1 Table. Dimensionless parameter values for the runs examined in the current paper are reported in Table 3. Particles were assumed to be rod-shaped with semi-major and semi-minor axes *a* = 1 μm and *b* = 0.5 μm. All computations were initialized using a single seed bacterium placed at the center of the bottom surface of the computational domain.

**Table 3:** Dimensionless parameter values for different computational cases examined. Cases include the ratio ${\dot{M}}_{E}/{\dot{M}}_{B}$ of EPS production rate to bacterial growth rate and the number *n*_*fim*_ of fimbriae per bacterial cell. (Case A-4 is the same as Case B-3).

Case	${\dot{M}}_{E}/{\dot{M}}_{B}$	*n*_*fim*_
A-1	0	1000
A-2	2	1000
A-3	4	1000
A-4	8	1000
B-1	8	0
B-2	8	100
B-3	8	1000
B-4	8	5000

### Reference Case (A-2)

**Fig 2:**
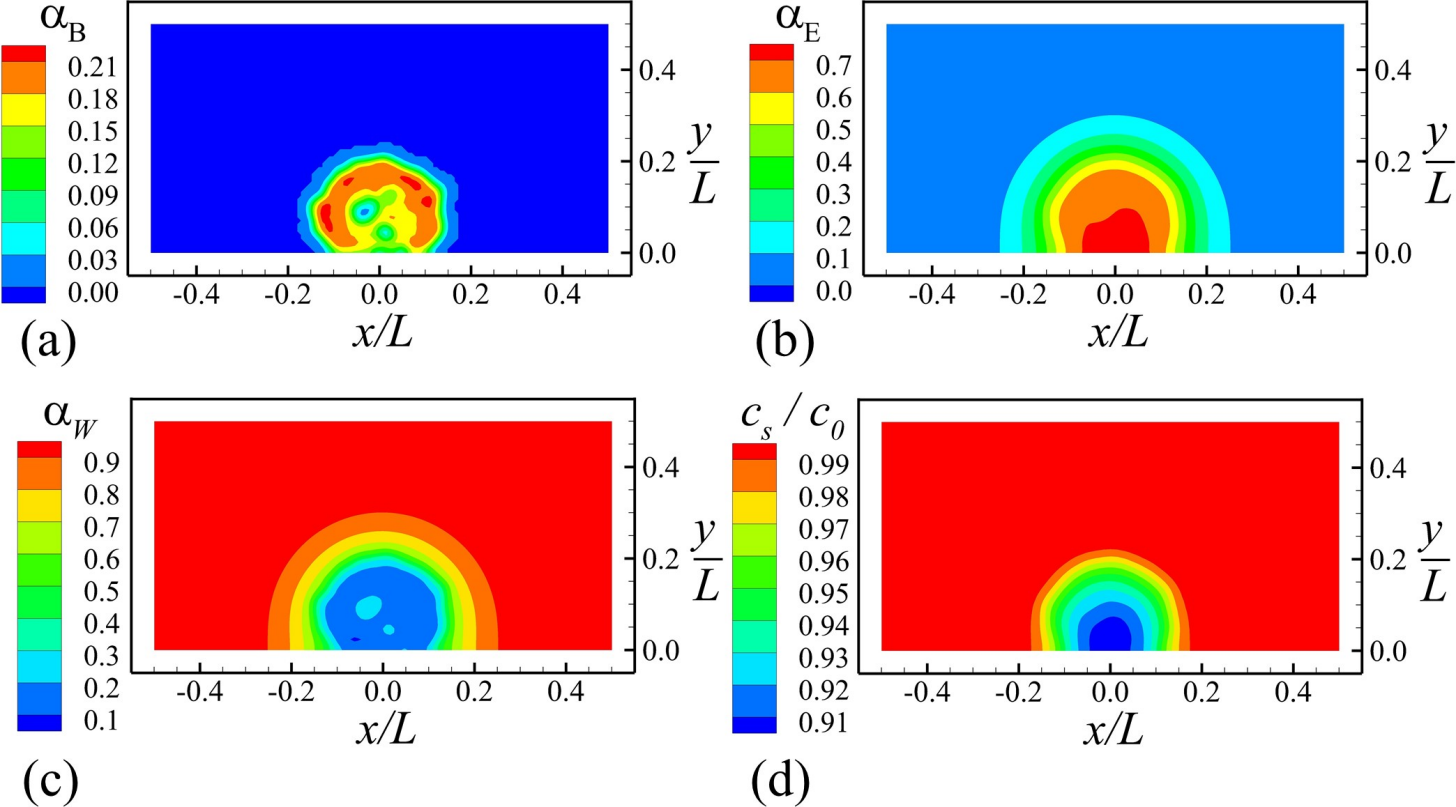
Slice plots at *z* = 0 of the bacterial colony. Plots show volume fractions of (a) bacterial cells, (b) EPS, (c) water, and (d) nutrient concentration, for Case A-2 when the total number of cells is around 5000. (Only bottom half of computational domain is shown).

A baseline computation (Case A-2) was conducted for a case with ${\dot{M}}_{E}/{\dot{M}}_{B}=2$ and *n*_*fim*_ = 1000, which is typical of common biofilm growth conditions. A bacterial colony grows from the seed cell in a roughly ball-like shape. Cross-sectional plots on the plane *z* = 0 are shown in Fig 2 at a time when the biofilm is well developed, showing the contour maps of the bacteria concentration *α*_*B*_, EPS concentration *α*_*E*_, water concentration *α*_*W*_, and nutrient mass concentration *c*_*S*_/*c*_0_. The bacterial colony forms a ball-like shape attached to the wall, with a higher concentration front near the outside of the ball where the nutrient and water availability is highest. Peak bacterial concentration is around 0.22 within the colony. The EPS is produced within the bacterial colony, but it is transported outward via both convection and diffusion, where iso-surfaces of the EPS concentration appear to have approximately hemispherical shapes. The EPS concentration approaches 0.7 within the colony. The water concentration decreases from nearly unity outside of the colony to around 0.1 within the colony. This strong reduction in water concentration is due to absorption of water by the bacteria in order to grow and produce EPS. A similar absorption occurs for the nutrients; however, the nutrient concentration reduces to only about 90% of its ambient value within the colony. The amount of nutrients required to produce a given about of biomatter is determined by the 'yield coefficient' $Y_{BS}\equiv -{\dot{M}}_{S}/({\dot{M}}_{B}+{\dot{M}}_{E})$, which was set equal to 0.1 in the current computations [64, 65].

**Fig 3:**
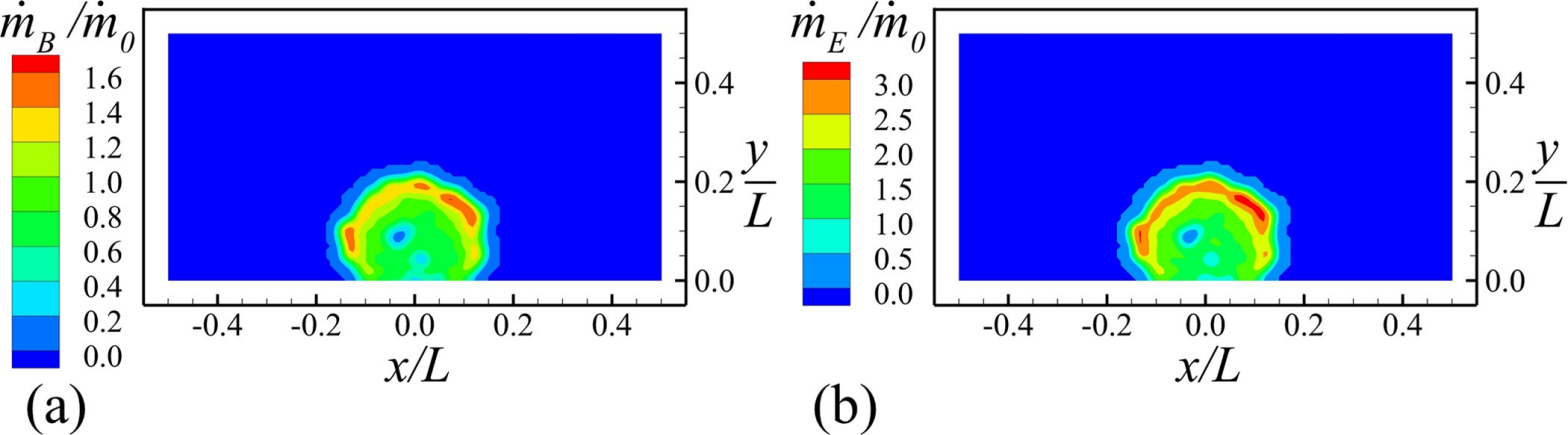
Slice plots at *z* = 0 showing the production rates (in ng/h). Plots show (a) bacterial cell (${\dot{m}}_{B}$) and (b) EPS (${\dot{m}}_{E}$) for Case A-2 when the total number of cells is around 5000. (Only bottom half of computational domain is shown).

**Fig 4:**
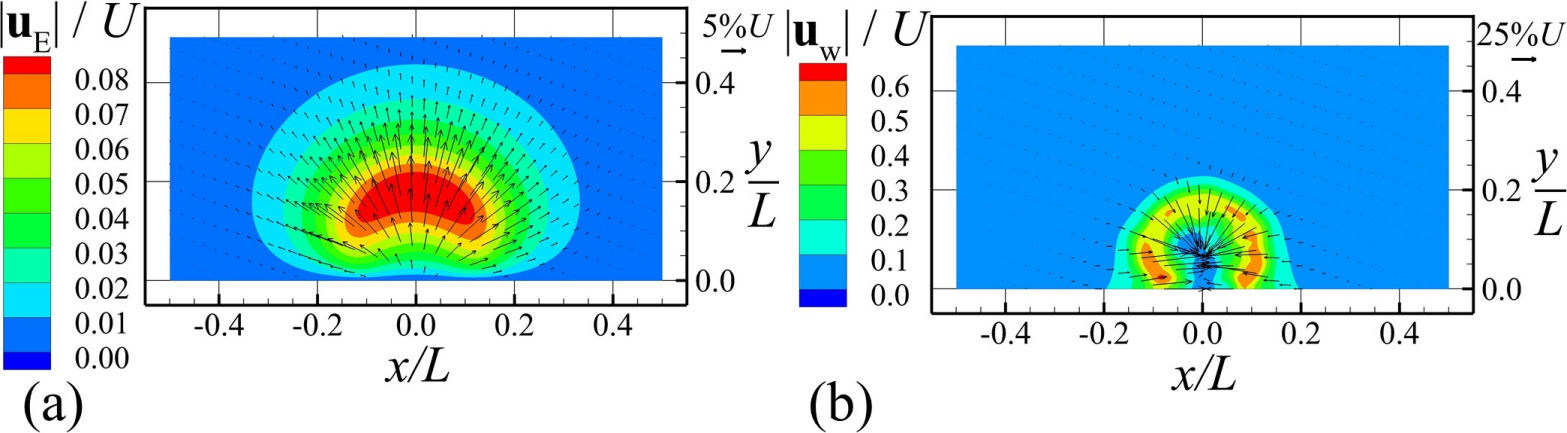
Slice plots at *z* = 0 showing magnitude of velocity fields and velocity components in the *x*- and *y*-directions. Plots show (a) EPS velocity field and (b) water velocity field for Case A-2 when the total number of cells is around 5000. The velocity components in the *x-* and *y-*directions are shown as vectors, and velocity magnitude is represented by contour plot. (Only the bottom half of computational domain is shown).

The rate of production of new cell material and EPS is shown in Fig 3. We see that both bacterial cell growth and EPS production are highest within a arched region near the outer surface of the colony, and that production ${\dot{M}}_{B}$ and ${\dot{M}}_{E}$ are both observed to decrease in the inner part of the colony due to shortage of both nutrients and water. The components of the EPS and water velocities are shown in Fig 4. The EPS velocity is oriented outward from the bacterial colony, and acts to push both EPS and bacterial cells away from the colony center. The water velocity field is of larger magnitude than EPS and generally oriented inward toward the bacterial cells. from both the top and sides of the colony.

### Sensitivity to EPS-to-bacteria production rate ratio

**Fig 5:**
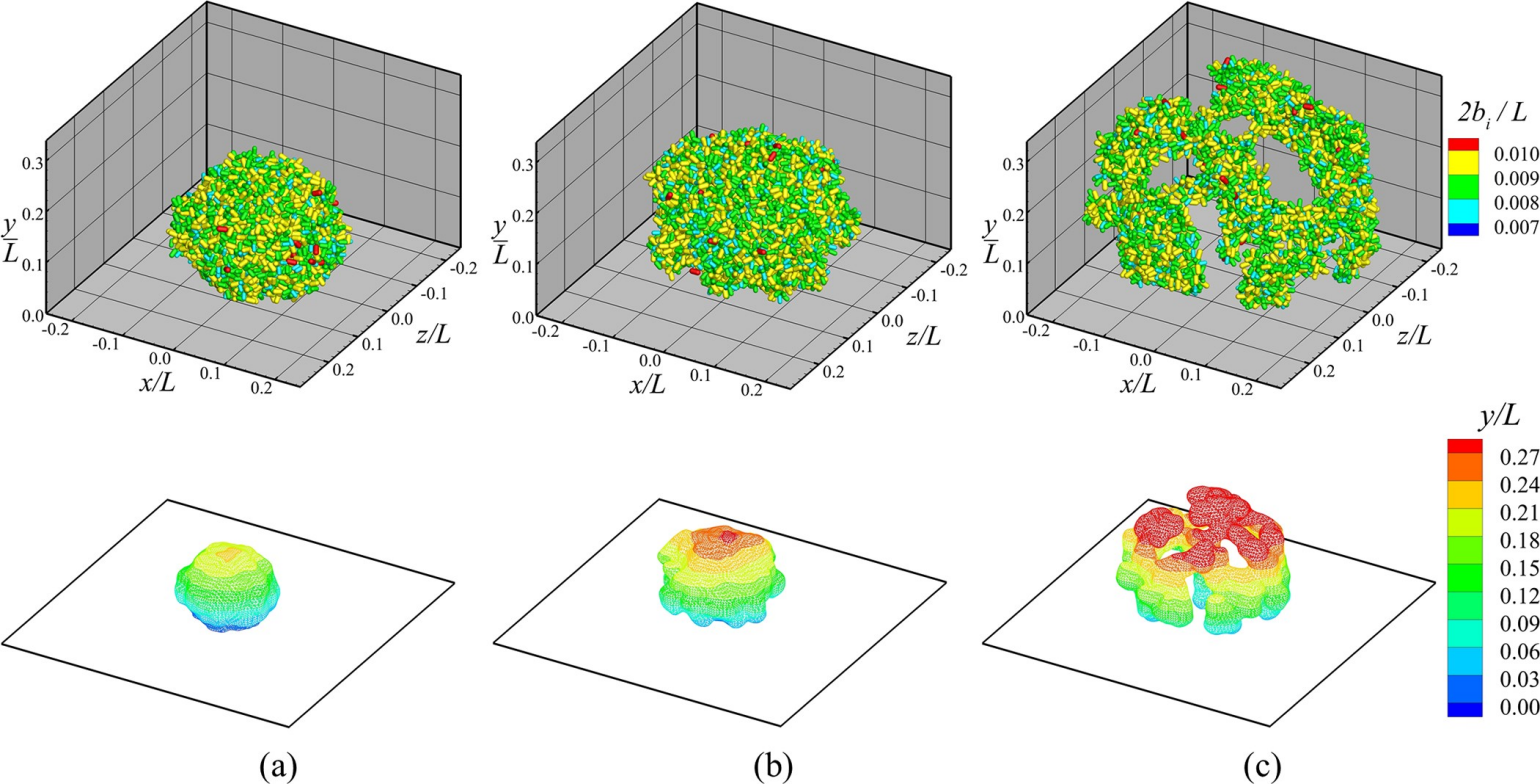
Comparison of bacterial colony structure for different values of ${\dot{M}}_{E}/{\dot{M}}_{B}$. Plots for cases (a) ${\dot{M}}_{E}/{\dot{M}}_{B}$ = 2 (Case A-2), (b) ${\dot{M}}_{E}/{\dot{M}}_{B}$ = 4 (Case A-3), and (c) ${\dot{M}}_{E}/{\dot{M}}_{B}$ = 8 (Case A-4), are captured when the total number of cells is around 5000. Top: Bacterial cells colored by their sizes. Bottom: The iso-surfaces of the EPS production rate ${\dot{m}}_{E}=1$ ng/h, with contour lines and colors to indicate the height above the *y* = 0 surface.

The significance of EPS on the biofilm growth is dependent on the EPS-to-bacteria growth rate ratio, defined by ${\dot{M}}_{E}/{\dot{M}}_{B}$. Examples with values of this ratio ranging from about 0.2–4.5 were recorded for different types of biofilms in Refs. [78–80], although values outside of this range are not atypical. The larger the value of this ratio, the more EPS is produced and the higher is the value of the EPS velocity magnitude during biofilm growth. Increase in EPS velocity magnitude results in an increase in outward cell drag force, and hence an increased tendency for the biofilm to break up and disperse. This tendency can be seen in Fig 5, which compares biofilm structure for three computations with ${\dot{M}}_{E}/{\dot{M}}_{B}$ values of 2 (Case A-2), 4 (Case A-3) and 8 (Case A-4). All other parameters are set the same as in the reference case discussed in the previous section. The top row of the figure shows the locations and orientations of the bacterial cells at a time when the number of cells equals approximately 5000 in each case. The cells are colored based on cell size. The bottom row of the figure gives the iso-surface ${\dot{m}}_{E}=1$ ng/h of the EPS production rate, with contour lines and colors to indicate the height above the *y* = 0 surface. For ${\dot{M}}_{E}/{\dot{M}}_{B}$ = 2, the bacterial colony is compact with a nearly axisymmetric shape. As the value of ${\dot{M}}_{E}/{\dot{M}}_{B}$ increases the colony becomes larger and more loosely structured, even though each run has the same number of cells at the time the figure was drawn. When ${\dot{M}}_{E}/{\dot{M}}_{B}$ = 8, the colony symmetry is broken and it adopts a complex shape with multiple nodes (or clusters) of cells connected by thinner strands.

**Fig 6:**
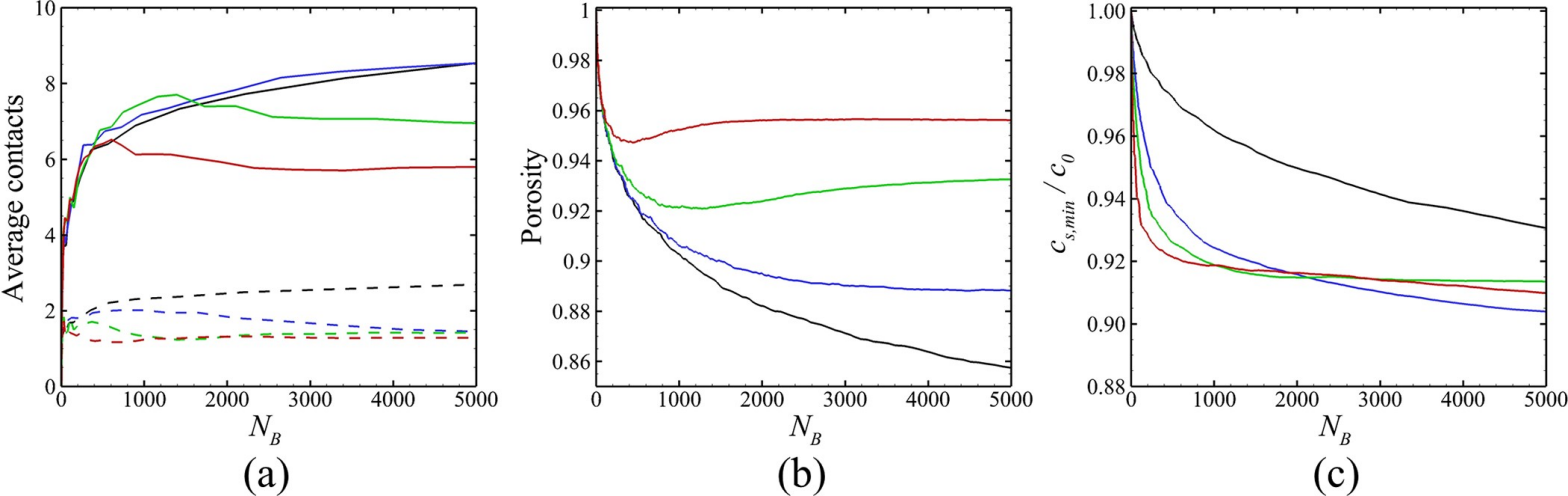
Variation as a function of cell numbers of various diagnostic parameters. The ratio ${\dot{M}}_{E}/{\dot{M}}_{B}$ for different cases are ${\dot{M}}_{E}/{\dot{M}}_{B}$ = 0 (black, Case A-1), 2 (blue, Case A-2), 4 (green, Case A-3), and 8 (red, Case A-4). Plots show (a) average number of fimbriae contacts (solid curves) and direct surface contacts (dashed curves) per bacteria, (b) porosity within the bacterial colony, and (c) minimum value of nutrient concentration *c*_*S*,min_/*c*_0_.

A number of parameters characterizing the biofilm development are plotted in Fig 6 as functions of the number of cells in the bacterial colony. The data compared in this figure has values of ${\dot{M}}_{E}/{\dot{M}}_{B}$ ranging from 0 to 8. Fig 6A shows the average number of contacts per bacterial cell, with fimbriae contacts indicated by solid lines and cell-cell surface contacts indicates by dashed lines. As might be expected, higher values of ${\dot{M}}_{E}/{\dot{M}}_{B}$ result in fewer of both types of contacts, since the particles become more separated as this ratio increases. The porosity within the bacterial colony is plotted in Fig 6B, which was computed by dividing one minus the volume of all particles by the volume of all grid cells that contain a particle. The porosity is observed to significantly increase as ${\dot{M}}_{E}/{\dot{M}}_{B}$ increases. When ${\dot{M}}_{E}/{\dot{M}}_{B}$ is small, the average number of contacts increases with the total number of bacterial cells and the porosity decreases with the total number of bacterial cells, in agreemnt with the measurement in Ref. [81]. Fig 6C plots the minimum value of the nutrient concentration within the colony divided by the ambient concentration, or *c*_*S*,min_/*c*_0_. The nutrient concentration within the bacterial colony is observed to decrease substantially with even a small amount of EPS production (between the ${\dot{M}}_{E}/{\dot{M}}_{B}$ = 0 and 2 cases), and then not to change much with further increase in ${\dot{M}}_{E}/{\dot{M}}_{B}$.

### Sensitivity to number of fimbriae per cell

**Fig 7:**
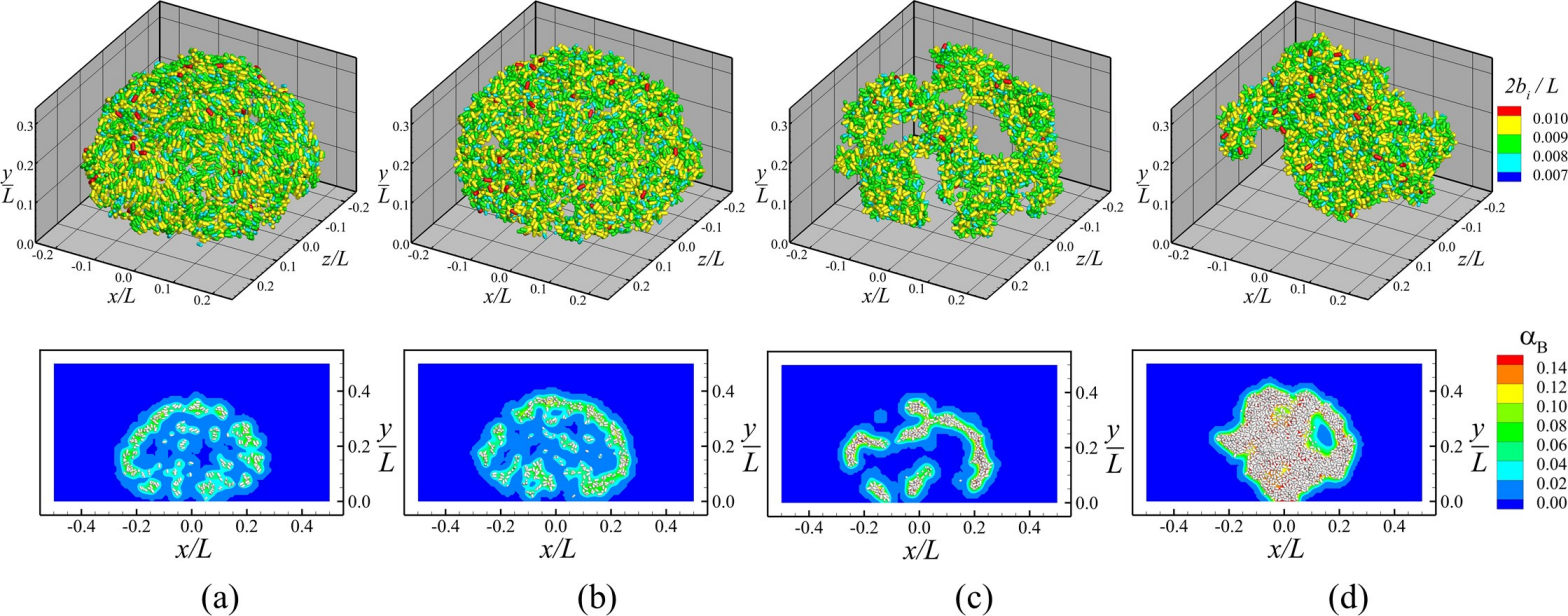
Comparison of bacterial colony structure for different values of fimbriae numbers per bacterial cell. Plots of cases (a) *n*_*fim*_ = 0 (Case B-1), (b) *n*_*fim*_ = 100 (Case B-2), (c) *n*_*fim*_ = 1000 (Case B-3), and (d) *n*_*fim*_ = 5000 (Case B-4), are captured when the total number of cells is around 5000. Top: Three-dimensional scatter plots with bacterial cells colored by their sizes. Bottom: Close-up slices in the *z* = 0 plane of the bacteria concentration *α*_*B*_. Particles shown in the lower plots lie in the region −0.01≤*z*/*L*≤0.01 surrounding the slice plane.

The role of fimbrial force on biofilm growth was examined using a series of computations in which the number of fimbriae per cell was increased in steps from 0 to 5000 (Cases B-1 thru B-4), with all other parameters being held the same. The computations were performed for a case with ${\dot{M}}_{E}/{\dot{M}}_{B}=8$, since we wanted to understand the effect of fimbrial force on the more loosely-structured biofilms typical of high EPS production rates. A comparison of the structure of the bacterial colony in the four computations at a time when the number of bacteria was approximately 5000 is shown in Fig 7, showing both a perspective 3-D view of the bacterial cells and a 2-D slice of the contours of bacteria concentration *α*_*B*_ in the *z* = 0 plane. These 2-D slices also show cells that lie within the region −0.01≤*z*/*L*≤0.01 surrounding the slice plane. This figure shows that increase in number of fimbriae causes several significant changes in the colony structure. In the case with no fimbriae (Case B-1), the colony has the shape of a slight compressed ball shape, extending to a height of Δ*y*/*L* = 0.39 and a width of Δ*x*/*L* = 0.47. The cells preferentially lie along the outer part of the colony, with a deficit in the bacteria concentration near the colony center. The cells themselves occur either in small clusters or singly, with neighboring cells having a strong tendency to align with each other. The addition of a small number of fimbriae in Case B-2 (with *n*_*fim*_ = 100) causes the colony to flatten more, with the width increasing to Δ*x*/*L* = 0.6 while the height remains approximately the same. The colony becomes asymmetrical when the fimbriae number per cell is increases to 1000 (Case B-3) and the fimbriae are observed to cluster into a small number of tightly-packed groups. For the largest value of fimbriae number examined (*n*_*fim*_ = 5000), the colony condenses into a tightly-packed mushroom shape, with a narrow base and a broader 'head'. In this structure, there is very little alignment of nearby particles with each other, but instead particles appear to be nearly randomly oriented.

**Fig 8:**
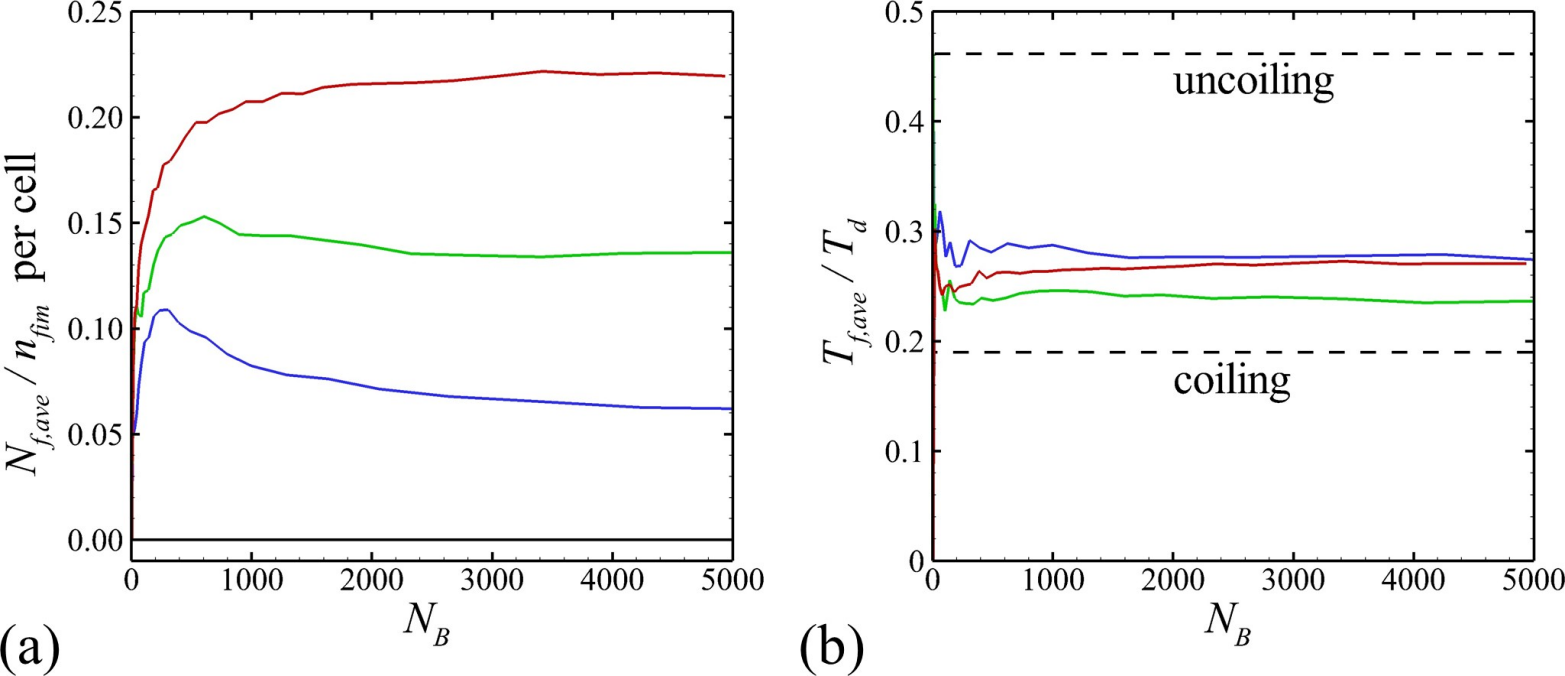
Plots showing diagnostics of fimbrial force as a function of number of bacterial cells. The numbers of fimbriae per bacterial cell for different cases are *n*_*fim*_ = 100 (blue, Case B-2), 1000 (green, Case B-3), and 5000 (red, Case B-4). Plots show (a) average number of attached fimbriae per cell, (b) average fimbriae tension per cell.

Fig 8A plots the average fraction of fimbriae per cell that are attached to other cells against the number of cells in the bacterial colony. This number increases rapidly early in the computation, but then appears to flatten out, and in several cases seems to approach an asymptotic value of between 10–25%. The fraction of attached fimbriae increases significantly with increase in total number of fimbriae, which is consistent with the observation that the fimbrial force makes the colony more tightly packed together. In Fig 8B, the average tension of one fimbria attachment is plotted as a function of number of cells. After some initial transients, this measure appears to remain approximately constant at between 25–30% of the detachment tension *T*_*d*_. It is noted that from the values given in Table 1 for uncoiling fimbriae is *T*_*uc*_/*T*_*d*_ = 0.46 and for coiling fimbriae is *T*_*c*_/*T*_*d*_ = 0.19, so this result suggested that some fimbriae are in a coiling state and others are in an uncoiling state.

**Fig 9:**
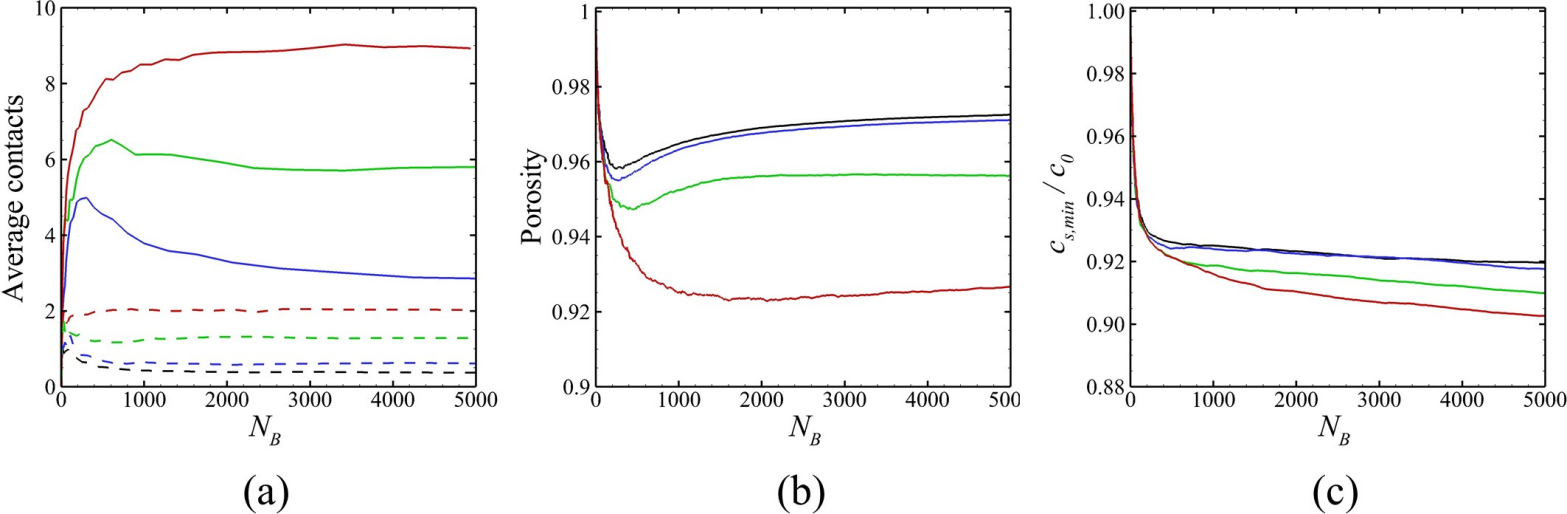
Variation as a function of cell numbers of various diagnostic parameters. The numbers of fimbriae per bacterial cell for different cases are *n*_*fim*_ = 0 (black, Case B-1), *n*_*fim*_ = 100 (blue, Case B-2), *n*_*fim*_ = 1000 (green, Case B-3), and *n*_*fim*_ = 5000 (red, Case B-4). Plots show: (a) average number of fimbriae contacts (solid curves) and direct surface contacts (dashed curves) per bacteria, (b) porosity within the bacterial colony, and (c) minimum value of nutrient concentration *c*_*S*,min_/*c*_0_.

Measures of the bacterial colony structure are plotted in Fig 9 as functions of number of bacterial cells. A very significant increase is observed in the number of fimbriae contacts per cell in Fig 9A, which more than doubles as the number of fimbriae is increased from 100 to 5000. The number of cell surface contacts also increases substantially, indicative of the bacterial colony becoming tighter packed by the increasing fimbrial force as *n*_*fim*_ increases. The bacterial colony porosity in Fig 9B decreases significantly as the number of fimbriae increases, again evidence that the cells within colony are becoming more tightly packed. In Fig 9C, we see that the minimum value of nutrient concentration is only slightly influenced by the number of fimbriae, suggesting that this parameter is primarily dependent on the number of cells and less sensitive to the colony structure.

**Fig 10:**
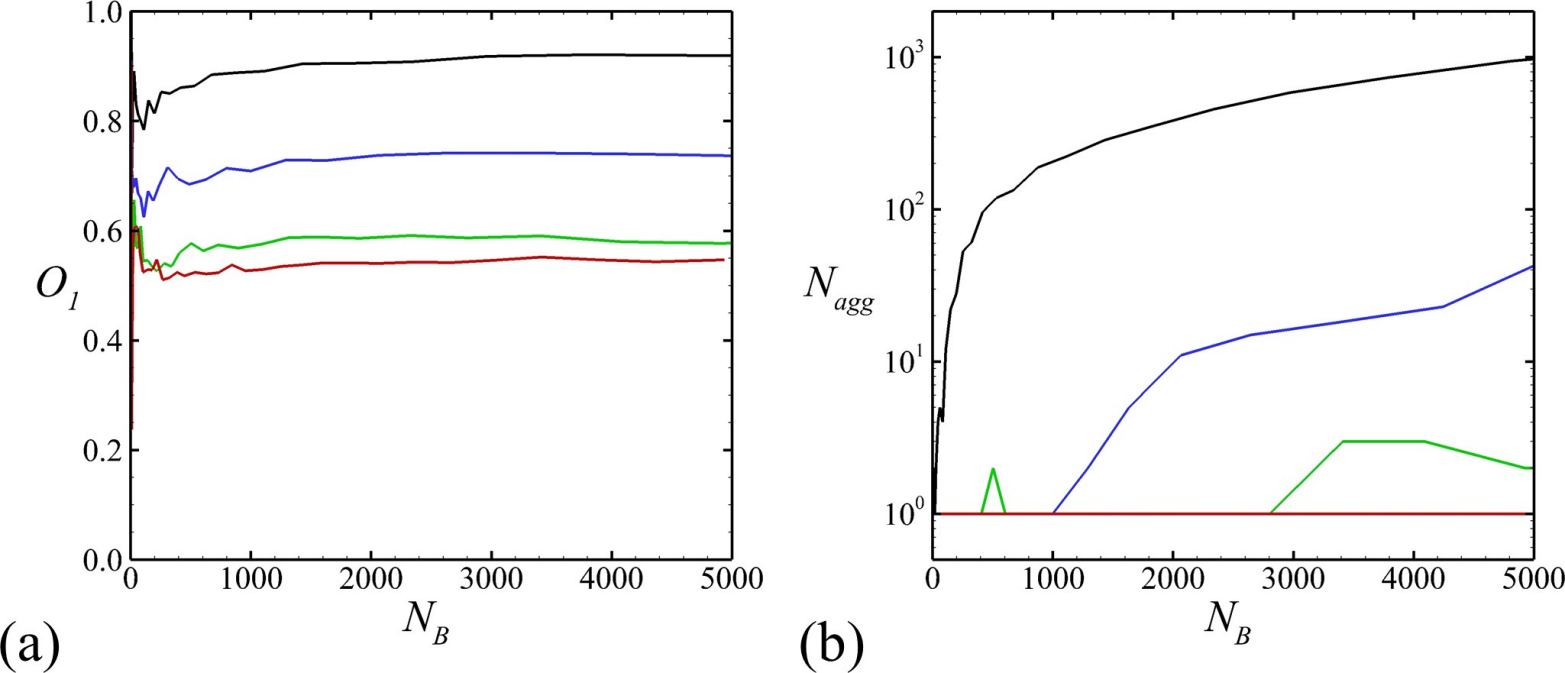
Variation as a function of cell numbers of two diagnostic parameters. The numbers of fimbriae per bacterial cell for different cases are *n*_*fim*_ = 0 (black, Case B-1), *n*_*fim*_ = 100 (blue, Case B-2), *n*_*fim*_ = 1000 (green, Case B-3), and *n*_*fim*_ = 5000 (red, Case B-4). Plots show (a) cell orientation parameter *O*_*I*_ and (b) number of agglomerates *N*_*agg*_.

A number of orientation measures were introduced by Chesnutt and Marshall [82] for characterizing alignment of particles in a cluster. In particular, *symmetry-axis-angle orientation measure O*_*I*_ was defined to relate the symmetry axis orientation of two contacting spheroidal particles, where *O*_*I*_ = 0 indicates that the symmetry axes are perpendicular and *O*_*I*_ = 1 indicates that the symmetry axes are parallel. Summing this measure over all contacting pairs of particles gives

$$O_{I}=\frac{1}{2N_{T}}\sum_{i=1}^{N}\sum_{j=1}^{N}a_{ij}\left|\cos\varphi_{ij}\right|,$$ (26)

where the *a*_*ij*_ equals unity if the particles are touching and zero otherwise, *N*_*T*_ is the number of touching particle pairs, and *N* is the number of particles. The time variation of *O*_*I*_ is plotted as a function of number of bacterial cells in Fig 10A, and observed to be nearly constant as the biofilm grows. However, the value of *O*_*I*_ decreases significantly as the number of fimbriae per cell is increased, changing from about 0.92 for the case with no fimbriae (Case B-1) to 0.56 for the case with *n*_*fim*_ = 5000 (Case B-4). This parameter provides a quantitative measure of the degree of alignment of nearby cells, and the observed decrease in this measure with *n*_*fim*_ is consistent with our previous qualitative observations that cells appear more randomly oriented and less aligned with each other as the fimbriae number increases. The reason for this behavior is that the fimbriae tension exerts a torque on cells in cases where the normal to the contact point of the fimbriae capsule with the cell surface does not pass through the cell center. This torque induces rapid rotation on a chain of particles that touch via the fimbriae connections, causing them to lose alignment with their neighboring particles. For all cases, *O*_*I*_ increases with the total number of bacterial cells after the initial random state, which indicates that the local orientation ordering increases at large numbers of cells. This observation is confirmed in the experimental observation in Ref. [81].

Fig 10B plots the number of agglomerates composing the bacterial colony as a function of the number of cells. An agglomerate is defined as an assemblage of particles in which each particle is in contact with at least one other particle in the assemblage, such that a continuous path between any two particles in the assemblage can be traced passing through these connected particles. Fig 10B is based on cell-cell surface contact, and not fimbriae contact. For the case with no fimbriae, the number of agglomerates in the colony is observed to increase with cell number, increasing to approximately 1000 agglomerates by the end of the computation. This behavior is characteristic of a very loose colony formed of dispersed clusters of particles that are held together by the EPS. Inclusion of even a small number of fimbriae changes this structure abruptly. For instance, in the case with *n*_*fim*_ = 100 (Case B-2), the colony is composed of a single agglomerate during the initial third of the computation, after which this agglomerate breaks up into 10–40 agglomerates during the latter two-thirds of the computation. For the case with *n*_*fim*_ = 1000 (Case B-3), the colony intermittently breaks up into 2–3 agglomerates and then reforms into a single agglomerate. The case with *n*_*fim*_ = 5000 (Case B-4) remains as a single agglomerate throughout the computation. Therefore, the local interaction between bacterial cells, such as fimbrial force, has significant influence on the structure and orientation of bacterial clusters, which is also reported for both experiments and modeling by Refs. [34, 43, 49].

**Fig 11:**
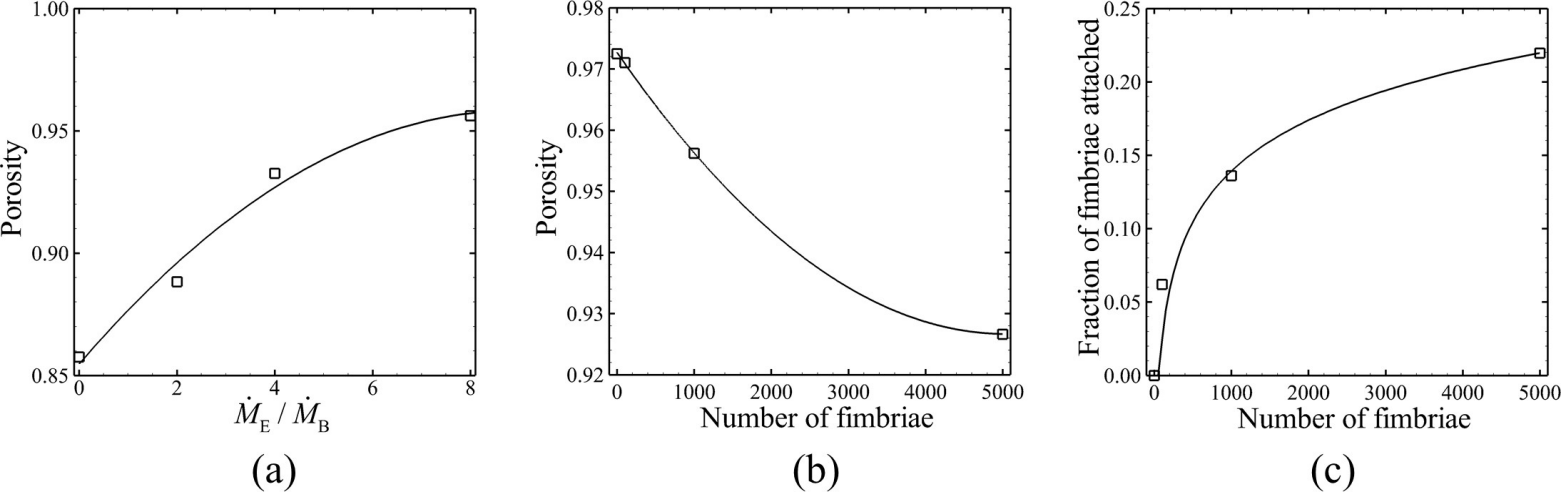
Steady state values of different parameters under different conditions. Plots showing long-time asymptotic values of (a) porosity at different growth rate ratio ${\dot{M}}_{E}/{\dot{M}}_{B}$, (b) porosity at different numbers of fimbriae per bacterial cell, and (c) average fraction of attached fimbriae at different numbers of fimbriae per bacterial cell. Solid lines indicate best-fit curves of quadratic form in (a) and (b) and logarithmic form in (c).

Fig 11A and 11B show the long-time asymptotic value of porosity as a function of growth rate ratio ${\dot{M}}_{E}/{\dot{M}}_{B}$ and number of fimbriae per bacterial cell. The porosity is observed to increase with increase in ${\dot{M}}_{E}/{\dot{M}}_{B}$ as the EPS flow causes the bacterial colony to expand, and the porosity decreases with increase in the number of fimbriae per cell as the fimbrial force causes the colony to contract. The lines in Fig 11A and 11B represent best quadratic fits to the data. Fig 11C shows the long-time asymptotic value of fraction of fimbriae that are attached to other cells as a function of the number of fimbriae per cell. The line in this figure is a logarithmic best-fit curve. The fraction of fimbriae attached is observed to increase rapidly with total number of fimbriae when this number is relatively small, varying from about *n*_*fim*_ = 0−1000, during which interval the colony is becoming increasingly compressed by the fimbrial force. As the total number of fimbriae becomes large, the elastic repulsion of the bacteria resist further compaction of the colony and the fraction of attached fimbriae is observed to be significantly less sensitive to changes in the total number of fimbriae.

## Conclusions

A hybrid computational method was developed for biofilm growth with cells of either spherical and rod-like (spherocylindrical) shapes. The model utilizes continuum mixture theory to simulate the different flow fields of water and EPS (as well as diffusion of nutrients, minerals and other chemicals through the water), while employing an adhesive discrete-element method to resolve interactions between individual bacterial cells. The continuum approach for water and EPS allows us to account for the important influences of osmotic pressure gradient, EPS viscous shear and EPS-water interfacial force, while the discrete simulation of individual cells allows us to incorporate important forces acting on the cells from drag due to motion of the cells relative to the EPS and as well as from forces such as lubrication, collision and adhesion forces between nearby cells.

Of particular focus in the current paper is the fimbrial force, in which the hair-like fimbriae appendages of one cell attach to a neighboring cell and exert a tensile force, as well as a related torque, on each attached cell. The fimbrial force is well known from experimental investigations to be of critical importance for biofilm development, but the role of fimbrial force on biofilm structural development has not been studied to date in the computational literature. We report on two related series of simulations designed to illuminate the competing influence of EPS drag and fimbrial force on a growing biofilm bacterial colony. The first computational set examines the significance of EPS flow on the bacterial colony by varying the ratio ${\dot{M}}_{E}/{\dot{M}}_{B}$ of EPS to bacterial production rate from 0 to 8. The bacterial colony is observed to transition from a single tightly-packed colony for small values of this ratio to an asymmetric structure with multiple nodes (or clusters) of cells, connected by thinner strands, for large values of this ratio. The second set of computations was designed to investigate the significance of fimbrial force for cases with relatively large values of ${\dot{M}}_{E}/{\dot{M}}_{B}$, by varying the number of fimbriae per cell from *n*_*fim*_ = 0 to 5000. These computations illustrate well the important role of the fimbriae in holding the bacterial colony together. With no fimbriae, the colony breaks up into small clusters of cells attached to each other by van der Waals surface adhesion, where all of these clusters are suspended in the biofilm by the EPS. As the fimbriae number is increased, these individual cell clusters coalesce into a single agglomerate.

Comparing with other simulation studies, our model captures the key advantages of both discrete and continuous biofilm growth models. It captures the effects of EPS osmotic spreading of biofilms under different growth rate ratios, as reported by Seminara et al. [41] and Yan et al. [83], and it reduces to a model similar to that Cogan & Keener [39] when the bacteria are restricted to move with the EPS. In the discrete part of the model, it confirms that cell number in a biofilm is a key parameter affecting colony structure. As the total cell number increases, both the number of cell contacts and the cell orientation match the qualitative trend described in experimental studies [81]. We have demonstrated that varying local interaction between individual cells, such as the total fimbriae number and EPS growth rate, can lead to qualitative change in the structural form of the bacterial colony. The overall shape of the bacterial colony observed in our simulations is similar to that noted in a number of other experimental studies [43, 49] and numerical analyses [34, 37, 42].

The current paper demonstrates that fimbrial force and cell drag associated with EPS production (and related relative flow of EPS past the cells) are significant effects that oppose each other during biofilm bacterial colony development. The ultimate structural form of a colony is largely dependent on the balance between these two competing effects.

## Supporting information

S1 Table Range and nominal values of various biofilm parameters. (References available from Jin et al. [44]).
(DOCX)
