# Supplementary material for: Mechanics of biofilms formed of bacteria with fimbriae appendages
Source: PLoS One. 2020 Dec 8;15(12):e0243280. doi: 10.1371/journal.pone.0243280 (PMC7723297; doi:10.1371/journal.pone.0243280)
Supplement: S1 Table — (References available from Jin et al. [44]). (DOCX) [file pone.0243280.s001.docx]

**S1 Table. Range and nominal values of various biofilm parameters. (References available from Jin et al.** [44]**)**

| **Parameter** | **Typical**  **Value** | **Range** | **Unit** |
| --- | --- | --- | --- |
| Bacteria cell diameter, $d_{B}$ | 0.5 | 0.1-2 | μm |
| Biofilm thickness, *L* | 100 | 20~200 | μm |
| Bacteria reproduction time, *T* | 3 | 1~10 | hr |
| Bacteria/water/EPS density, $\rho$  | 10^3^ | - | kg/m^3^ |
| Water viscosity, $\mu_{W}$ | 10^-3^ | - | Pa⋅s |
| EPS viscosity, $\mu_{E}$ | 10^4^ | 10^2^~10^7^ | Pa⋅s |
| Interaction coefficient, $\Phi$ | 10^12^ | 10^11^~10^14^ | Pa⋅s/m^2^ |
| Osmotic pressure factor, $\Gamma$ $\psi(\alpha_{E})=\Gamma\alpha_{E}^{2}(\alpha_{E}-\alpha_{E,ref})$   | 10^3^ | 4300 | N/m^2^ |
| Nutrient diffusion coefficient, $D_{S}$ | 100 | 50~400 | μm^2^/s |
| Maximum growth rate, $M_{B,0}$ | 1/T | - | hr^-1^ |
| Monod saturation constant, $K_{S}$ | 10^-4^ | 10^-5^~10^-3^ | kg/m^3^ |
| Maximum nutrient concentration, $c_{S0}$ | 10^-1^ | 10^-3^~10^-2^ | kg/m^3^ |
| Growth yield coefficient, $Y_{BS}$ | 0.1 | 0.045~0.44 | - |
| Bacteria elastic modulus, *E_B_* | 10^3^ | 10~10^5^ | N/m^2^ |
| Characteristic velocity *U* | 10 |  | nm/s |
| Characteristic changing rate 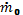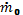 | 1.8 |  |  |
